# Cost‐effectiveness of personalized omalizumab dosing for chronic spontaneous urticaria

**DOI:** 10.1111/ced.15316

**Published:** 2022-09-19

**Authors:** Sarah Denman, Helin Smith, Gururaj Arumugakani, Anoop Mistry, Sinisa Savic

**Affiliations:** ^1^ Department of Clinical Immunology and Allergy St James's University Hospital Leeds Yorkshire UK; ^2^ Leeds Institute of Rheumatic and Musculoskeletal Medicine University of Leeds Leeds UK

## Abstract

The licensed dose for omalizumab within Europe for chronic spontaneous urticaria (CSU) is 300 mg every 4 weeks, and is based on the most effective dose identified in clinical trials. However, many patients require longer‐term treatment with omalizumab and there is limited guidance on how to manage these patients. We report on a large cohort of 357 patients with CSU who have been treated over a 10‐year period on a personalized dosing regimen. Our results showed a 4% reduction in drug cost for this personalized dosing regimen compared with having all patients on the standard regimen of omalizumab 300 mg every 4 weeks. In addition, by increasing the dose, we were able to treat 22% of patients more effectively, using the principle aim of zero CSU symptoms; prior to this regimen, these patients had been achieving only partial response. Omalizumab doses and frequency should be adjusted depending on clinical response to allow for improved benefits for both patients and healthcare systems.

## Graphical abstract

Omalizumab doses and frequency should be titrated to clinical response. There was a 4% reduction in drug cost for our personalized dosing regimen. Updosing allowed 22% current cohort to be treated more effectively.

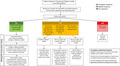

In 2014, the European Medicines Agency (EMA) approved the use of omalizumab in patients with chronic spontaneous urticaria (CSU) at a dose of 300 mg every 4 weeks. This was based on the most effective dose identified in clinical trials.^1^, ^2^ At the time, the data on longer‐term use beyond 6 months were limited, and the product literature continues to state this. As a result, funding arrangements for continuous omalizumab treatment varies considerably between countries. In the UK, the National Institute for Health and Care excellence guidelines on omalizumab state that in patients who have responded, treatment should be stopped after six doses.^3^ However, omalizumab does not alter the natural course of CSU, and our experience is that many patients need treatment beyond 6 months because their symptoms have not gone into remission^4^ (Table 1). There is limited guidance on how to manage these patients in the longer term. In addition, there are patients who fail to respond to standard doses. These patients can be challenging to manage if immunosuppression has failed or they may need longer term immunosuppression, which is associated with increased risks. The EAACI/GA^2^LEN/EuroGuiDerm/APAAACI urticaria guidelines recommend dose increases of omalizumab in those patients not responding to standard doses.^5^

**Table 1:** Patient demographics.

Parameter	Cohort	
	Full	Current^a^
Patients, *n*	357	170
Sex, *n* (%)		
Male	93 (26)	45 (26)
Female	264 (74)	125 (74)
Age, years		
Mean	44	45
Range	18–89	19–89
Omalizumab duration, months		
Mean	33	50
Range	2–134	7–134

^a^ Assessed November 2021.

Within our centre, omalizumab for CSU was introduced in September 2010, prior to the licensing approval. We successfully use a system whereby patients have their doses titrated up or down based on the degree of symptom control (Fig. 1). In order to assess the cost‐effectiveness and clinical benefits of this pathway, we retrospectively reviewed patients started on omalizumab over a 10‐year period between September 2010 and September 2020.

**Figure 1:**
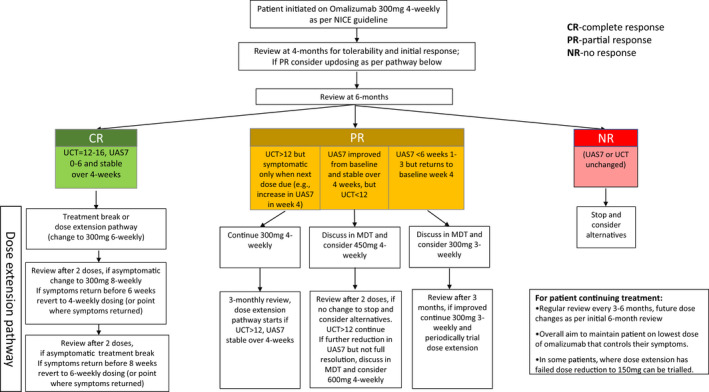
Personalized dosing pathway. [Colour figure can be viewed at wileyonlinelibrary.com]

## Report

In total, 357 patients received omalizumab for CSU (Table 1). During their treatment course, 130 patients (36%) were managed on lower doses, while 77 (22%) required higher doses and 7 (2%) had both higher and lower doses at different stages in their treatment pathway. Those on active treatment with omalizumab as of November 2021 were subsequently analysed separately. At the time of analysis, there were 70 patients (41%) on the standard dose of 300 mg every 4 weeks, 63 patients (37%) on a lower dose and 37 patients (22%) on a higher dose (Table 2). The drug cost per annum of this cohort was calculated on the current dose. There was a 4% reduction in drug cost for our personalized dosing regimen compared with having all patients on the standard dose of 300 mg every 4 weeks for CSU.

**Table 2:** Breakdown of dosages of current cohort.

Dosage	Patients, *n*
150 mg every week	2
150 mg every 6 weeks	1
150 mg every 7 weeks	1
300 mg every 2 weeks	1
300 mg every 3 weeks	15
300 mg every 4 weeks	70
300 mg every 5 weeks	13
300 mg every 6 weeks	21
300 mg every 7 weeks	9
300 mg every 8 weeks	17
300 mg every 9 weeks	1
450 mg every 3 weeks	5
450 mg every 4 weeks	10
450 mg every 5 weeks	1
450 mg every 6 weeks	1
600 mg every 4 weeks	2

The overall aim of treatment is no symptoms.^5^ If standard doses were used, 22% of our current cohort would have had only a partial response or would have needed to switch to immunosuppression. The latter can be associated with increased adverse effects, especially if required longer term. In addition, immunosuppression requires regular blood monitoring, at a financial cost to the healthcare system. Patients with poorly controlled CSU require more frequent review appointments. Conversely, those with longer dose intervals need less frequent reviews. These factors support the conclusion that personalized dosing allows more patients with CSU to be treated effectively in a pathway that is also more cost‐effective (Fig. 1).

There are only limited studies in the literature reporting how patients with CSU are managed on omalizumab in the longer term and most of these have been performed outside the UK.^4^, ^6^, ^7^ Our pathway is most similar to that described by Giménez Arnau *et al*.,^8^ who also concluded that even patients who do not initially respond to treatment can obtain significant reductions in disease activity if treatment is continued for up to 24 weeks. This is something we have witnessed in practice, with patients appearing to be nonresponders until beyond the fourth dose.

Our pathway uses two main types of patient‐reported outcome measures (PROs), namely the Urticaria Control Test (UCT) and the 7‐day Urticaria Activity Score (UAS7). Other PROs may be considered on an individual patient basis. The UCT provides insight into the overall level of CSU control and can be completed within an outpatient appointment. The UAS7 provides more detailed information and more importantly, variance over a longer period, e.g. the UCT may indicate poor control but review of the UAS7 may show that the patient is asymptomatic for the first 3 weeks before having their symptoms return in Week 4. Using both PROs provides a better overall picture of the patient's response to omalizumab and helps to guide treatment decisions.

In complete responders, there is a patient/clinician decision at 6 months as to whether to have a treatment break or gradually extend the interval between omalizumab doses. For some patients, especially those with significant anxiety about stopping treatment or those who have had a difficult journey to complete remission, the latter is often more psychologically acceptable. At any point during the interval extension, patients can revert to the most recent effective dose if there is an increase in symptoms, with regular review and a new plan made to try extending the interval again after a mutually agreed time frame. In some patients, we have tried reductions to 150 mg per dose but this can be difficult to manage if symptoms return before the 4‐week interval, and so is not routinely included in our pathway.

Dose increases (450/600 mg) for partial response are discussed by the team, revisiting symptoms to ensure no alternative diagnoses have been missed and considering alternative treatments. In some patients the effects of omalizumab diminish before 4 weeks, and in these patients reducing the interval between doses to the time at which symptoms return can be highly effective.

In our review we found that a small number of patients (*n* = 7) had moved between both lower and higher doses during their treatment pathway. Fluctuations in dose, even within the same individual, support the natural fluctuation of CSU itself. Although some omalizumab dose predictors (e.g. body mass indicator, previous ciclosporin use, age), the evidence is limited.^6^, ^9^ An increased understanding of the natural CSU fluctuations and biomarker variability may be needed to see if this is transferable to dosing.

For patients who need to continue treatment longer term, the overall aim is to maintain them on the lowest effective dose with regular dose reduction reviews, especially if UAS7 score is 10 and/or UCT is 16. This supports the recommendation of the EAACI/GA^2^LEN/EuroGuiDerm/APAAACI urticaria guidelines on stepping down treatment, and is similar to the principles used for biologics for other conditions.^5^

To conclude, our large cohort indicates that personalized dosing of omalizumab in patients with CSU is a more cost‐effective pathway, which results in more patients achieving improved response.

**Learning points**

• Omalizumab doses and frequency should be adjusted depending on clinical response.
• There was a 4% reduction in drug cost for our personalized dosing regimen compared with a standard regimen.
• Increasing the dose allowed 22% of the current cohort to be treated more effectively.

## Conflict of interest

SD and SS have received speaker fees from Novartis; the fees to SD were paid into the departmental education and training account.

## Funding

None.

## Ethics statement

Ethics approval not applicable. The patient provided informed consent for publication of their case details and images.

## Data Availability

Data are available on request from the corresponding author.
